# Perspectives on the prospective development of stroke-specific lower extremity wearable monitoring technology: a qualitative focus group study with physical therapists and individuals with stroke

**DOI:** 10.1186/s12984-020-00666-6

**Published:** 2020-02-25

**Authors:** Dennis R. Louie, Marie-Louise Bird, Carlo Menon, Janice J. Eng

**Affiliations:** 1 Graduate Program in Rehabilitation Sciences, Faculty of Medicine, University of British Columbia, 212-2177 Wesbrook Mall, Vancouver, BC V6T 1Z3 Canada; 2 Rehabilitation Research Program, GF Strong Rehabilitation Research Lab, Vancouver Coastal Health Research Institute, 4255 Laurel Street, Vancouver, BC V5Z 2G9 Canada; 3 Department of Physical Therapy, Faculty of Medicine, University of British Columbia, 212-2177 Wesbrook Mall, Vancouver, BC V6T 1Z3 Canada; 4 School of Health Sciences, College of Health and Medicine, University of Tasmania, Locked Bag 1322, Launceston, TAS 7250 Australia; 5 Menrva lab, Schools of Mechatronic Systems and Engineering Science, Simon Fraser University, Metro Vancouver, BC V5A 1S6 Canada

**Keywords:** Stroke, Fitness tracker, Wearable electronic devices, Walking, Remote sensing technology

## Abstract

**Background:**

Wearable activity monitors that track step count can increase the wearer’s physical activity and motivation but are infrequently designed for the slower gait speed and compensatory patterns after stroke. New and available technology may allow for the design of stroke-specific wearable monitoring devices, capable of detecting more than just step counts, which may enhance how rehabilitation is delivered. The objective of this study was to identify important considerations in the development of stroke-specific lower extremity wearable monitoring technology for rehabilitation, from the perspective of physical therapists and individuals with stroke.

**Methods:**

A qualitative research design with focus groups was used to collect data. Five focus groups were conducted, audio recorded, and transcribed verbatim. Data were analyzed using content analysis to generate overarching categories representing the stakeholder considerations for the development of stroke-specific wearable monitor technology for the lower extremity.

**Results:**

A total of 17 physical therapists took part in four focus group discussions and three individuals with stroke participated in the fifth focus group. Our analysis identified four main categories for consideration: 1) ‘Variability’ described the heterogeneity of patient presentation, therapy approaches, and therapeutic goals that are taken into account for stroke rehabilitation; 2) ‘Context of use’ described the different settings and purposes for which stakeholders could foresee employing stroke-specific wearable technology; 3) ‘Crucial design features’ identified the measures, functions, and device characteristics that should be considered for incorporation into prospective technology to enhance uptake; and 4) ‘Barriers to adopting technology’ highlighted challenges, including personal attitudes and design flaws, that may limit the integration of current and future wearable monitoring technology into clinical practice.

**Conclusions:**

The findings from this qualitative study suggest that the development of stroke-specific lower extremity wearable monitoring technology is viewed positively by physical therapists and individuals with stroke. While a single, specific device or function may not accommodate all the variable needs of therapists and their clients, it was agreed that wearable monitoring technology could enhance how physical therapists assess and treat their clients. Future wearable devices should be developed in consideration of the highlighted design features and potential barriers for uptake.

## Background

Individuals with stroke commonly face mobility limitations, beginning at stroke onset [1] and continuing past discharge into the community [2], and demonstrate a range of gait deviations due to altered motor control and resulting compensatory movement patterns [3]. Improving walking quality and quantity is a major focus of therapy [4], as doing so can improve mobility, fitness, quality of life, and prevent secondary complications [5, 6]. One avenue to target walking for individuals with stroke may be to utilize wearable monitoring technology, as previous research has shown that application of an activity monitor can improve user self-efficacy and physical activity levels in various patient populations including older adults, breast cancer survivors, and those with chronic obstructive pulmonary disease [7–11]. Additionally, wearable monitors have been increasingly utilized by therapists and researchers to assess various outcomes relating to exercise and physical activity, [12, 13] within therapy and between visits, to ensure exercise targets are met [14].

The majority of currently available wearable monitoring technology has not been developed specifically for stroke-related impairments and movement patterns. For example, consumer activity monitors are often limited by a minimum walking speed or movement amplitude in order to provide accurate and reliable feedback [15, 16]. Research efforts have attempted to adapt available wearable monitoring technology to meet the needs of individuals with stroke with increasing accuracy, from simple solutions such as wearing hip-situated fitness trackers at the ankle [17, 18], to developing software algorithms to analyze captured data to recognize movements patterns specific to stroke [19–21]. The advances in wearable monitoring have reached a point at which designing stroke-specific wearable monitoring technology is a realistic priority to assess outcome and enhance rehabilitation interventions [22].

Much of the efforts to design stroke-specific wearable monitoring technology has so far focused on the hemiparetic upper limb [23–26]. This is unsurprising, as many individuals with stroke report long-term upper limb deficits or disability [27], and upper limb recovery has been identified as a top research priority from the perspective of individuals with stroke and their health professionals [28]. Conversely, limited efforts have been made in applying sensing technology to design stroke-specific wearable monitors for the hemiparetic lower limb. Research has shown that accelerometry can be reliable and valid in measuring physical activity after stroke [29], and new technologies to quantify foot pressure, leg motion, and muscle activity are being shown to be applicable to stroke [30, 31]. Thus, there is a gap in wearable monitoring technology for individuals with stroke, between what can be designed to improve rehabilitation of the lower extremity and what is currently available.

In order to develop devices that fill this niche, it is important to involve end-users in the development process from the onset to ensure initial efforts are relevant to the individuals who will ultimately use them, [32, 33] which inevitably are individuals with stroke and their physical therapists. This user-centered design approach is optimal for identifying relevant factors and technical aspects that should inform design choices [32, 33]. Thus, the objective of the current study was to identify important considerations in the future development of stroke-specific lower extremity wearable monitoring technology for rehabilitation, from the perspective of physical therapists and individuals with stroke.

## Methods

This study involved focus groups mainly with physical therapists who work closely with individuals with stroke. Focus groups were chosen as they are able to rapidly generate information on collective views [34], which may be useful in the initial phase of research and development (e.g., of interventions, questionnaires, technology) [35]. A qualitative descriptive approach was utilized in order to gain rich description from physical therapist experience and perceptions [36]. The final focus group was conducted with individuals with stroke. All participants provided written informed consent and were offered a small honorarium for their time to participate.

### Participants

#### Therapists

A convenience sample of practicing physical therapists was recruited from a local rehabilitation hospital and two private neurorehabilitation physical therapy clinics. Therapists were eligible if they were 19 or older and had at least 1 year of experience working with individuals with stroke. Therapists were recruited through e-mail distribution of a study information letter by contacting the manager at each respective site. A total of 17 therapists were recruited to participate in four focus groups (Table 1).

**Table 1:** Demographic characteristics of the participants in the five focus groups (physical therapists, individuals with stroke), at the time of inclusion

**Focus group 1: Public health care physical therapists (*n*= 5)**	
Sex, male/female	1/4
Age, median (range), years	42 (28–49)
Professional experience with stroke, median (range), years	8 (5.5–18)
**Focus group 2: Private practice physical therapists (*n*= 4)**	
Sex, male/female	0/4
Age, median (range), years	30.5 (28–51)
Professional experience with stroke, median (range), years	5.5 (2–25)
**Focus group 3: Public health care physical therapists (*n*= 4)**	
Sex, male/female	0/4
Age, median (range), years	36 (26–42)
Professional experience with stroke, median (range), years	6.25 (2.5–21)
**Focus group 4: Private practice physical therapists (*n*= 4)**	
Sex, male/female	1/3
Age, median (range), years	29.5 (27–32)
Professional experience with stroke, median (range), years	3.75 (1.5–9)
**Focus group 5: Individuals with stroke (*n*= 3)**	
Sex, male/female	2/1
Age, median (range), years	55 (47–62)
Time since stroke onset, median (range), years	1.25 (1–3.5)
Currently using technology for own health (n)	
Yes	2
No	1

#### Participants with stroke

Individuals with stroke were recruited purposively from a list of previous research participants discharged from the local rehabilitation hospital. The participants with stroke were required to be ambulatory in the community, at least 6 months post-stroke with leg weakness which affects walking, over the age of 19, and able to communicate verbally and freely in English. Three individuals with stroke were recruited for the final focus group (Table 1).

### Procedures

Focus groups lasted between 45 and 60 min, with three to five participants in each. Focus groups with stroke clinicians took place at their respective workplace. A quiet conference room away from other staff or patients was utilized within the rehabilitation hospital and within each private clinic. For the final focus group conducted with individuals with stroke, a conference room in the aforementioned rehabilitation facility was used.

Focus groups were conducted between June 2018 and September 2018 by a moderator (DRL) in the presence of an observer (MLB). The moderator guided the discussion, while the observer took notes of the conversation and occasionally asked clarifying questions to further explore a discussion point. The moderator used semi-structured focus group interview guides, the development of which was informed by the Technology Acceptance Model [37]. The Technology Acceptance Model can be broadly applied to various technologies and has been previously extended to wearable fitness technology [38]; it highlights variables, such as perceived usefulness and ease of use, that inform user attitudes which eventually influence technology adoption. The interview guides were refined through extensive discussions with qualitative research experts, therapists, and engineers. An audio recording device was used at each focus group to record the conversation for later transcription and analysis.

For physical therapists, the moderator led a discussion surrounding their experience working with people with stroke to improve their leg function and walking, as well as their perspective towards the role of wearable monitoring technology for this purpose. They were then asked broad questions regarding the features of a prospective device, without specifying a type of device they should envision. In addition, they were asked questions regarding their own perceptions towards integrating technology into clinical practice. For participants with stroke, the moderator led a discussion about their perspective towards wearable technology designed to detect movement specific to stroke, as well as whether it would be helpful for their day-to-day routine. The focus group guides for physical therapists and for participants with stroke are available in Appendix 1 and 2, respectively.

Audio recordings of focus groups were transcribed verbatim and checked for accuracy. Participants were assigned an alphanumeric identification code to anonymize transcript data and excerpts included in this manuscript; physical therapist participants were given the identifier P#, and participants with stroke were given the identifier S#.

### Research team and reflexivity

DRL is a male PhD student with prior experience conducting semi-structured interviews and a licensed physical therapist in the area of neurorehabilitation. This allowed him to connect with fellow physical therapists in order to adequately moderate and explore the focus group discussion. However, one focus group was conducted at a previous place of employment, which may have influenced how therapists responded. MLB is a female faculty member in physical therapy, with previous experience in focus group facilitation and qualitative research. Personal assumptions and reflections were discussed between the two members conducting focus groups before and during data collection and analysis. CM is a male professor in mechatronic engineering with a research interest in biomedical technology. JJE is a female professor in physical therapy with extensive research experience in clinical intervention development.

### Data analysis

The focus group data were analyzed inductively using qualitative content analysis [39–41]. Transcripts of the focus groups were read, re-read, and coded independently by two of the investigators (DRL, MLB). Patterns of meaning were identified, allowing for primary codes to be generated. Through iterative discussion and consultation amongst all investigators, codes were consolidated and grouped together to establish sub-categories, which were then checked against each of the original transcripts. In the final stage, the sub-categories were combined into broader groups to form categories. Hand-written notes from the moderator and observer during the focus groups were also consulted.

The primary means for ensuring trustworthiness was through triangulation, reflexivity, and peer debriefing. Conducting a focus group with individuals with stroke to corroborate or contrast with clinician perceptions served as a form of data source triangulation [42]. Meetings between the focus group moderator and observer throughout the data collection process to compare interview notes and to discuss expected and unexpected tangents facilitated reflexivity. Having multiple investigators independently code transcripts and compare codes through peer debriefing was a form of investigator triangulation and encouraged reflection on and refinement of categories as they were formulated [42].

## Results

Four overarching categories were formed from the focus groups regarding important considerations in the development of future stroke-specific lower extremity wearable monitoring technology, presented below. The categories and their sub-categories are listed in Table 2.

**Table 2:** Categories and sub-categories derived from content analysis

Category	Sub-categories
Variability	• Variability of clients • Therapy considerations • Therapy approaches • Focus of therapy
Context of use	• Usage location • Usage purpose • Non-representative performance (clinic vs. home)
Crucial design features	• Facilitators of adopting technology • Helpful measurements • Ideal design (usability and wearability) • Multimodal/customizable feedback
Barriers to adopting technology	• Shortcomings of current technology • Cost a limiting factor • Barriers to integrating technology • Concerns for future technology

### Variability

Physical therapists highlighted the variability they encounter when working with individuals with stroke, from the range in patient presentation after stroke to the techniques and approaches they employ during rehabilitation. For example, a physical therapist in the rehabilitation facility (P3) commented on the composition of their caseload, “it can be people that are very high level, a lot of fine tuning … and then sometimes we do get the very low level that haven’t walked but are now starting to show some recovery … so it can be quite a spectrum.” Another physical therapist (P14) commented that they see a “mixed bag” of patients and ability in their private practice setting. Importantly, one participant with stroke (S3) also highlighted the variability in access to health care depending on a patient’s geography and financial status.

With regard to the variable nature of therapeutic approach, therapists make many considerations in planning their treatment, depending on “if they have good sensation … good proprioception, good sense of mid-line, good trunk control (P12).” Despite the many variables of stroke rehabilitation, therapists agreed that their treatment approach was dependent on client goals, which frequently includes a focus on the leg or walking:

“I feel [treatment is] guided by client goals … of course walking is one of their main goals, so we always kind of try to divide priorities between their hand and leg … in my opinion the body is a unit, unless you get the leg strong you can’t have the hand work and vice versa, so you never literally stop working on one portion.” – P6

When considering the applicability of a stroke-specific wearable device to their practice and clients, the therapists expressed concern that it would not work for everyone. One physical therapist (P15) suggested that “it depends on the person and their goals” and that some of their clients “would probably like it and some probably wouldn’t use it at all”, depending on factors such as motivation and adherence. Another therapist (P17) alluded to the potential discrepancy between the utility of a wearable device and the complexity of rehabilitating walking, that “it’s such a multi-factorial [issue] … there’s so many different reasons why they may not be achieving their walking goals” and that “finding the significance of the data is the hard part”. A potential solution to this was suggested by a colleague (P15), that “the option to select different things would be nice.”

### Context of use

A category that was formed from the focus groups surrounded the context of use for stroke-specific wearable monitoring technology. Therapists discussed at length the exact nature of wearable monitoring technology that would benefit their clients with stroke, including the potential usage setting, purpose, frequency, and operator. Some therapists gravitated towards a potential assessment tool that could be used in the clinic to enhance rehabilitation by accurately measuring various aspects of leg impairment and movement, such as compensatory movements or muscle activation. A physical therapist (P6) commented, “the priority would be for the clinicians to use it first … to correlate the feedback with what you find clinically”. Another therapist (P2) similarly stated, “because the gait cycle can go fast … sometimes I’m just not sure what I’m getting with my hands … if you get that data back maybe then you can actually … see what you can focus on”.

On the other hand, other practitioners preferred a device that could be given to their clients to wear between therapy sessions, as P4 described, “something that can also tell me how much someone’s doing outside of therapy time”. Both therapists and individuals with stroke frequently discussed the potential to measure the difference between stroke clients’ in-clinic performance and their actual performance at home or in the community. One individual with stroke commented:

“I always wanted to impress my physical therapist, and she said, ‘I know you’re not gonna walk a snail’s pace as soon as you turn the corner ‘cause she knew me’ – to get that perfect gait you had to walk so slow, when she knew as soon as I was out of her sight I would be like, zoom!” – S3

Several therapists indicated that it would be of value to know how much activity their clients are achieving on their own, as well as the quality of their movement. They would prefer to check a device periodically to monitor the effects of their therapy on home performance, instead of using an in-clinic tool. Another physical therapist (P10) stated, “monitoring how they are in treatment and what they do outside, I’m more interested about, versus assessment”. Another individual with stroke (S2) echoed that such a device could be “used at your therapy place, and then you can go home and take it from there as well yourself.” Therapists and individuals with stroke agreed that home monitoring could improve the wearer’s motivation to be more physically active at home or in the community.

Physical therapists mostly foresaw a device that they operated, whether in clinic or for home monitoring. Very few comments arose regarding a stroke-specific device that was independently used by individuals with stroke for the purpose of tracking their own fitness. One therapist (P12) commented on their experience with providing specific data to clients, “often these numbers are somewhat meaningless to them. They go, ‘I can see the improvement, but what does that actually mean?’” This exact sentiment was echoed in the focus group with individuals with stroke:

“I don’t even want that information. I want somebody who knows what they are doing to tell me ‘you are not doing this, here is what you have to do to fix that’, ‘cause I will screw myself up quite happily”. – S1

### Crucial design features

Participants in each focus group listed many considerations and features that could be incorporated into a prospective wearable monitoring device. A multitude of desired measurements was suggested, from joint range of motion and muscle activation, to temporospatial features of gait and symmetry of weightbearing. Physical therapists envisioned an ideal stroke-specific wearable monitor that could do anything from providing a kinematic breakdown of the wearer’s gait, to capturing compensatory movement strategies and toe clearance. Participants with stroke emphasized specific muscle activation and timing of activation as their primary measures of interest above and beyond step counts and movement speed. For example, one of the individuals with stroke (S3) stated, “measuring steps is one thing, but I mentioned that I’m also working on my gait.”

In addition to the specific measurements that therapists desired, the analysis revealed several key design features relating to wearability, usability, and functioning that participants considered necessary for stroke-specific wearable monitoring technology. Therapists and individuals with stroke alike agreed that the prospective device should be small, inconspicuous, and lightweight. It should be easily applied and operated with the use of one hand, and as one of the individuals with stroke described (S3), “it has to be pretty much fool proof.” In a similar vein, physical therapists highlighted the importance of user-friendliness; P5 stated, “easy to set-up, and taking all that information off the computer … very user friendly, that’s a really good feature for a device.” For physical therapists, understanding the data that is returned was another important design feature – whether data were returned relative to expected norms or processed in another way:

“If there was something out there that could collect more information that we could integrate, like maybe understanding the information, that would be more valuable than just collecting.” – P2

Other crucial aspects of a potential stroke-specific device were minimal or quick calibration, programmable interface with a smart phone or laptop, and ease of data access. A comprehensive list of suggested features and measures is included in Table 3.

**Table 3:** Summary of suggested measures, functions, and design features (usability, wearability) of prospective wearable monitoring technology

**Measures**	**Functions**
• Muscle activation detection	• Video-pairing
• Gait measurements	• Bluetooth connectivity
- Timing of muscle activation	• Detects bilaterally
- Compensatory pattern detection	• Integrate data with medical records
- Heel contact	• Ability to manipulate data
- Temporospatial features	• Remote programming and data access
- Kinematic features	• Raw and processed data
- Toe trajectory	• Comparison to normative data
- Stride length	• Continuous feedback/readings
• Heart rate monitoring	• Biofeedback
• Weight bearing	- Audio (multiple pitches)
• Symmetry	- Lights (colour-coded)
• Differentiate activation vs. tone	- Vibration
• Differentiate concentric vs. eccentric	- On handheld device
• Force and loading	- Numeric feedback
• Step counts	- Customisable (volume, cues)
**Usability**	**Wearability**
• Desired variable selection	• Small size, inconspicuous
• Reproducible set-up	• Lightweight
• Phone interface	• Cosmetically pleasing
• Laptop interface	• Simple disinfection
• Simple/reusable calibration	• Donning/doffing with one arm
• Malfunction alert	• Visual cue at wrist
• Turns on upon application	
• Usable with one hand	
• Customisable app	
• Good battery life	

Therapists in every focus group agreed upon the importance of integrating purposeful biofeedback into future wearable technology for stroke. Beyond returning numerical data to either therapists or individuals with stroke, therapists envisioned live feedback that could act as an intervention to improve wearer performance. One therapist (P17) foresaw the use of feedback as a way to provide continuity between the clinic and community, stating, “maybe with a device, they can learn to use it … and then it gives that feedback that you did it like you did in therapy.” Various forms of feedback were suggested, including haptic vibration, visual light display, audio beeping, and even remote vibration on a handheld device. One concern that was raised in the discussion of purposeful biofeedback was the timing of feedback, and whether the wearer was alerted to correct or incorrect movements. Another physical therapist (P8) suggested the option for customizable feedback: “I like the idea of vibrating feedback, and maybe the handheld device vibrates when there’s a mistake … maybe have the option of both.”

Therapists were understanding that many of their desired measurements are likely not feasible or cannot all be integrated into a single device. As such, therapists offered several suggestions for ensuring successful adoption of future wearable technology and highlighted ease of use, minimal specialized training, and consistent usage as key criteria for a novel device to be considered for acquisition. Above all, regardless of which measurements a novel wearable device is capable of capturing, each therapist focus group tended to agree that the technology should be unique and provide meaningful information:

“They’d have to offer something different than what’s existing … like what do they offer that’s different and why do I want one over the other. What’s the easiest for my brain to use, because we have a lot of things on our plate.” – P12

### Barriers to adopting technology

Physical therapists in all focus groups discussed concerns and barriers that could limit the uptake of stroke-specific wearable monitoring technology. Drawing from their experience with current technology, they described design flaws with current and prospective technology that limit their relevance or utility in therapy. For example, one physical therapist (P9) stated, “some things can take too long to set up … then you’ve wasted their time and yours.” Another therapist (P4) echoed this sentiment, that despite the useful information garnered from one of their specialized devices, it is “very time-consuming to set up and take down, and also quite tedious doing the editing of the data … so for that reason it doesn’t get pulled out a lot.” Concerns over accuracy and calibration were also raised, that device reliability or an involved calibration process could deter therapists. Pain, discomfort, and skin sensitivity were also brought up by therapists, as well as participants with stroke, as obvious reasons to avoid using wearable technology.

A common concern identified by participants with stroke and physical therapists was the potential cost of future wearable monitoring technology, as well as who should pay for the device. The conversation around cost was intertwined with the discussion around how the prospective technology would be used. If used purely as an in-clinic assessment device, physical therapists would purchase the technology depending on its price. However, for at-home monitoring purposes, focus groups had differing perspectives. For example, a participant in the stroke focus group (S3) stated, “I see this as an assistive device for [physical therapists] so I think it should be more on a lending basis”. In contrast, physical therapists had conflicting opinions. One therapist (P16) stated, “if they were taking it home, I would say they would have to be purchasing it”, while another therapist (P17) expressed concern over the financial burden of stroke and the potential for their clinic to absorb the cost.

Physical therapists also alluded to administrative and infrastructural reasons that limit adoption of current and new technology into stroke rehabilitation. One such administrative barrier is infection control policy, captured in the following exchange between two hospital physical therapists:

“Anything we’re using within the therapy gym, if it’s not a client’s own it has to be … the infection control guidelines have become so much more strict in the last five years, I’d say. So things have to be single patient, it has to be really easy to clean otherwise.” – P2

“They want us to even stop using transfer belts because of infection control, so anything that needs to be strapped to a limb, like a transfer belt, they want us to get away from using.” – P3

Other infrastructural practice barriers that influence physical therapists’ decision to utilize technology include limited resources, including both time and space. One physical therapist (P13) described the extra time commitment to using specialized equipment, “an important consideration is the time to put a therapist to interpret this data … to do all the charting and all the forms.” Another therapist (P12) in the same group shared, “I think it gives useful information, but the training and the fact that it’s cumbersome makes it not as desirable.” An additional administrative concern is data safety, especially around devices with Internet connectivity. The same physical therapist, who has worked in a practice leadership role, mentioned, “the only practicality that just needs to be considered is that we don’t always have Wi-Fi in public healthcare spaces, and then there’s lots of privacy firewall things.”

Physical therapists themselves were sometimes the barrier to integrating technology within their practice. Therapist attitudes, biases, and assumptions were apparent and potentially limiting to the use of technology. For example, one therapist (P9) associated the use of technology for therapy with a reduction in participation by their patients, stating that their patients are “sometimes more passive with technology, they’re not as actively engaged in rehab if they have something that’s assisting them.” One of their colleagues (P6) expressed a similar concern about developing a reliance on technology if over-utilized, that “it has to be used in the right proportion so that they don’t get dependent on it, but it still benefits them”. Another therapist commented on the tendency for practitioners to be overwhelmed by the sheer amount of technology options to incorporate into practice:

“There’s so many different reasons to have different things, some staff just get more comfortable with a few items and don’t branch beyond that … a lot of personal motivators that hold people back or dictate if they’re gonna use something.” – P2

Another sentiment that arose was the notion that technology quickly becomes obsolete, and that therapists would sometimes prefer to rely on their own skills. Despite these barriers, the overall sentiment across the focus groups was one of cautious acceptance:

“I’m not against technology, I find it practically quite useful – quite difficult to use sometimes just for all those reasons.” – P12

## Discussion

The objective of this study was to identify important considerations for the development of prospective stroke-specific lower extremity wearable monitoring technology for rehabilitation, from the perspective of physical therapists and individuals with stroke. Following user-centered design, physical therapists with specialized knowledge of the mobility needs of individuals with stroke and the principles of stroke rehabilitation were involved to bridge the gap between technical design and clinical utility; individuals with stroke with lived experience of altered walking ability and participation in rehabilitation were also involved as a key stakeholder group. To the best of our knowledge, this is the first study to investigate either clinician or client perspectives regarding stroke-specific wearable monitoring technology for the lower extremity. The analysis identified four key categories of considerations for engineers and researchers seeking to develop wearable technology for stroke rehabilitation that will enhance uptake: Variability, Context of use, Crucial design features, and Barriers to adopting technology.

### Variability

The notion of variability in stroke rehabilitation, whether in therapeutic approach or in patient presentation, is important to accept when beginning to develop wearable technology, as it is unlikely that any single device or function will be useful to all therapists and their clients. In a similar qualitative study in which the authors explored the perceptions of therapists and people with stroke on robotic devices for the upper extremity, one theme revolved around the heterogeneity of arm impairment and therapist focus as a challenge to developing new devices [43]. However, regardless of this challenge of variability, the potential benefit of developing stroke-specific wearable monitoring technology was voiced by all participants in our study. With the understanding that future wearable monitoring technology will not appeal or be applicable to all end-users, whether therapist or client, developers can then streamline their focus on designing devices with a specific purpose and target demographic.

### Context of use

Physical therapists foresaw useful application of wearable technology to enhance their practice for either assessment within the clinic, or for home monitoring and feedback of performance; similarly, the participants with stroke envisioned a device that their physical therapist operated, despite being the wearer. This resonates with the findings from previous reviews of wearable technology for rehabilitation. Shull et al. [22] described the proliferation of wearable sensors and feedback devices and highlighted the future potential for home monitoring devices to capture performance in natural human environments and for continuous, long-term monitoring and intervention. With regard to the upper extremity, Maceira-Elvira et al. [44] suggested that offering home-based therapy, monitored remotely by therapists, has the potential to improve rehabilitation outcomes by allowing individuals with stroke to train in a familiar environment.

With advances in sensing technologies, prospective devices could potentially be designed for assessment to assist therapists in performing clinical measurements that were previously inaccessible or difficult to do on their own. For example, efforts have been made to embed electromyography into socks and shirts, in the form of smart textiles [45, 46]; additionally, monitoring systems involving multiple sensors are now available that can detect the slightest change in balance or gait behaviours as a result of rehabilitation [47]. Therapists in our study expressed excitement over the many ways in which wearable monitoring technology for assessment could help with their ability to target standing, balance, and gait compensations. This is in line with previous research showing that physical therapists and individuals with stroke welcome incorporating research technology for the clinical assessment for balance and mobility [48], and presents a less frequently explored potential of wearable monitoring technology for prospective development.

### Crucial design features

Physical therapists and individuals with stroke listed a plethora of measures and design features for developers to consider for incorporation in future wearable monitoring technology. As the potential to develop wearable technology that can capture the even the finest details of leg function and walking becomes a reality, considerations of the design features that will facilitate adoption are key. From the perspective of wearable product design for individuals with a disability, usability and wearability are essential factors that should inform development [49, 50]. While a wearable monitor may be developed to carry out a specific measurement, usability reflects whether the device is user-friendly, including easy to set-up and minimal errors [49]. On the other hand, wearability are those features that make a wearable monitor actually acceptable to put on, including aesthetics, ease of donning and doffing, and comfort.

Several functions were proposed to enhance the utility of wearable activity monitors. In addition to measuring outcome data, features such as remote data access, processed data and comparisons to the norm, as well as having customizable feedback modalities, enhanced the allure of prospective wearable monitoring technology. Physical therapists were excited for the prospect of potentially using remotely programmable wearable technology to facilitate telehealth, a growing focus to increase accessibility to health care services in remote areas through the use of digital communication services [51]. Regardless of what measurements prospective wearable monitoring technology can achieve, it is the additional design features that will determine its adoptability.

### Barriers to adopting technology

Many concerns raised relating to the adoption of technology were the converse flaws of the design features suggested for future devices. Characteristics that would detract from usability and wearability included difficult set-up, discomfort, prolonged calibration, and other flaws that could ultimately stymy the uptake of future devices. Other research on the development of wearable devices for stroke or other populations list similar design flaws to this effect [43, 52, 53]. Thus, for the development of any future wearable monitoring technology, it is important to thoroughly consider the ways in which a device may or may not be usable or wearable.

Some therapists’ resistance to using technology in rehabilitation, such as the belief that technology may not necessarily improve the outcomes or participation from clients, may also influence uptake of future technology. This belief is not unfounded, as a recent Cochrane review of studies utilizing commercial activity monitors in the stroke population to improve physical activity concluded that there is insufficient evidence to support the use of activity monitors to increase physical activity after stroke [54]. However, the wearable monitors included in the review were not designed for stroke, and outcomes may differ if the technology itself were developed to target the specific goals, needs, and concerns of individuals with stroke.

### Limitations

The major limitation of this study was the lack of participants with stroke, with only three individuals with stroke forming a single focus group. While the findings from their focus group largely supported the considerations put forward by therapists, it is possible that further focus groups with this stakeholder group may have yielded divergent opinions or led to the development of different overarching categories. Though physical therapists may have the extensive knowledge and experience in the field of stroke rehabilitation, individuals with stroke are ultimately the ones to wear any future monitoring devices and so more individuals should have been recruited for this study. Future studies should position individuals with stroke as the primary end-user of wearable monitoring technology and should extensively explore their perspectives towards designing future wearable monitoring technology, whether for therapists to enhance rehabilitation or for personal use.

Additionally, physical therapists participated in focus groups within their work environment and amongst their colleagues, which may have affected their willingness to share any thoughts that were contradictory to others despite opposing responses and debate being encouraged. Each focus group was comprised of therapists from the same workplace, and so discussion between therapists working in different practice settings, with patients of different acuity, may have been missed.

## Conclusion

Stroke-specific lower extremity wearable monitoring technology is viewed positively by clinicians and individuals with stroke. While a single, specific device or function may not accommodate all the variable needs of therapists and their clients, it was agreed that wearable monitoring technology could enhance how physical therapists assess and treat their clients. Future wearable devices should be developed with intentional consideration of the setting and purpose of the device usage, design features including usability and wearability, and potential barriers for uptake. Prospective prototypes should be tested with physical therapists and individuals with stroke as the next step in the development process.

## Supplementary information

**Additional file 1.** Focus group guide for healthcare professionals.

**Additional file 2.** Focus group guide for individuals with stroke.

## Data Availability

Audio files and transcripts are not available for review because of the risk of participant identification.

## References

[1] Paolucci S, Bragoni M, Coiro P, De Angelis D, Fusco FR, Morelli D (2008). Quantification of the probability of reaching mobility independence at discharge from a rehabilitation hospital in nonwalking early ischemic stroke patients: a multivariate study. Cerebrovasc Dis.

[2] Wesselhoff S, Hanke TA, Evans CC (2018). Community mobility after stroke: a systematic review. Top Stroke Rehabil.

[3] Balaban B, Tok F (2014). Gait disturbances in patients with stroke. PM R.

[4] Harris JE, Eng JJ (2004). Goal priorities identified through client-centred measurement in individuals with chronic stroke. Physiother Can.

[5] Han P, Zhang W, Kang L, Ma Y, Fu L, Jia L (2017). Clinical evidence of exercise benefits for stroke. Adv Exp Med Biol.

[6] Wein T, Lindsay MP, Côté R, Foley N, Berlingieri J, Bhogal S (2018). Canadian stroke best practice recommendations: secondary prevention of stroke, sixth edition practice guidelines, update 2017. Int J Stroke.

[7] Gualtieri L, Rosenbluth S, Phillips J (2016). Can a free wearable activity tracker change behavior? The impact of trackers on adults in a physician-led wellness group. JMIR Res Protoc.

[8] Larsen RT, Christensen J, Juhl CB, Andersen HB, Langberg H (2019). Physical activity monitors to enhance activity in older adults – a systematic review and meta-analysis. Eur Rev Aging Phys Act.

[9] Nguyen NH, Hadgraft NT, Moore MM, Rosenberg DE, Lynch C, Reeves MM, Lynch BM (2017). A qualitative evaluation of breast cancer survivors’ acceptance of and preferences for consumer wearable technology activity trackers. Support Care Cancer.

[10] McMahon SK, Lewis B, Oakes K, Guan W, Wyman JF, Rothman AJ (2016). Older adults’ experiences using a commercially available monitor to self-track their physical activity. JMIR Mhealth Uhealth.

[11] Qiu S, Cai X, Wang X, He C, Zügel M, Steinacker JM (2018). Using step counters to promote physical activity and exercise capacity in patients with chronic obstructive pulmonary disease: a meta-analysis. Ther Adv Respir Dis.

[12] Wright SP, Hall Brown TS, Collier SR, Sandberg K (2017). How consumer physical activity monitors could transform human physiology research. Am J Phys Regul Integr Comp Phys.

[13] Allouch SB, van Velsen L (2018). Fit by bits: an explorative study of sports physiotherapists’ perception of quantified self technologies. Stud Health Technol Inform.

[14] Connell LA, Klassen TK, Janssen J, Thetford C, Eng JJ (2018). Delivering intenstive rehabilitation in stroke: factors influencing implementation. Phys Ther.

[15] Schaffer SD, Holzapfel SD, Fulk G, Bosch PR (2017). Step count accuracy and reliability of two activity tracking devices in people after stroke. Physiother Theory Pract.

[16] Fulk GD, Combs SA, Danks KA, Nirider CD, Raja B, Reisman DS (2014). Accuracy of 2 activity monitors in detecting steps in people with stroke and traumatic brain injury. Phys Ther.

[17] Klassen TD, Semrau JA, Dukelow SP, Bayley MT, Hill MD, Eng JJ (2017). Consumer-based physical activity monitor as a practical way to measure walking intensity during inpatient stroke rehabilitation. Stroke..

[18] Klassen TD, Simpson LA, Lim SB, Louie DR, Parappilly B, Sakakibara BM (2016). “Stepping up” activity poststroke: ankle-positioned accelerometer can accurately record steps during slow walking. Phys Ther.

[19] Chia Bejarano N, Ambrosini E, Pedrocchi A, Ferrigno G, Monitcone M, Ferrante S (2015). A novel adaptive, real-time algorithm to detect gait events from wearable sensors. IEEE Trans Neural Syst Rehabil Eng.

[20] Hsu WC, Sugiarto T, Lin YJ, Yang FC, Lin ZY, Sun CT (2018). Multiple-wearable-sensor-based gait classification and analysis in patients with neurological disorders. Sensors (Basel).

[21] Lopez-Meyer P, Fulk GD, Sazonov ES (2011). Automatic detection of temporal gait parameters in poststroke individuals. IEEE Transf Inf Technol Biomed.

[22] Shull PB, Jirattigalachote W, Hunt MA, Cutkosky MR, Delp SL (2014). Quantified self and human movement: a review on the clinical impact of wearable sensing and feedback for gait analysis and intervention. Gait Posture.

[23] Fanchamps MHJ, Selles RW, Stam HJ, Bussmann JBJ (2018). Development and validation of a clinically applicable arm use monitor for people after stroke. J Rehabil Med.

[24] Held JPO, Luft AR, Veerbeek JM (2018). Encouragement-induced real-world upper limb use after stroke by a tracking and feedback device: a study protocol for a multi-center, assessor-blinded, randomized controlled trial. Front Neurol.

[25] Sadarangani GP, Jiang X, Simpson LA, Eng JJ, Menon C (2017). Force myography in individuals with stroke with mild to moderate upper-extremity impairments: a preliminary investigation in a controlled environment. Front Bioeng Biotechnol.

[26] Simpson LA, Mow A, Menon C, Eng JJ (2019). Preliminary examination of the ability of a new wearable device to capture functional hand activity after stroke. Stroke..

[27] Dromerick AW, Lang CE, Birkenmeier R, Hahn MG, Sahrmann SA, Edwards DF (2006). Relationships between upper-limb functional limitation and self-reported disability 3 months after stroke. J Rehabil Res Dev.

[28] Pollock A, St George B, Fenton M, Firkins L (2014). Top 10 research priorities relating to life after stroke – consensus from stroke survivors, caregivers, and health professionals. Int J Stroke.

[29] Gebruers N, Vanroy C, Truijen S, Engelborghs S, De Deyn PP (2010). Monitoring of physical activity after stroke: a systematic review of accelerometry-based measures. Arch Phys Med Rehabil.

[30] Jiang X, Chu KHT, Khoshnam M, Menon C (2018). A wearable gait phase detection system based on force myography techniques. Sensors (Basel).

[31] Wang C, Kim Y, Shin H, Min SD (2019). Preliminary clinical application of textile insole sensor for hemiparetic gait pattern analysis. Sensors (Basel).

[32] Redström J (2006). Toward user design? On the shift from object to user as the subject of design. Design Stud.

[33] Gulliksen J, Göransson B, Boivie I, Blomkvist S, Persson J, Cajander Å (2003). Key principles for user-centred systems design. Behav Inform Technol.

[34] Gill P, Stewart K, Treasure E, Chadwick B (2008). Methods of data collection in qualitative research: interviews and focus groups. Br Dent J.

[35] Stalmeijer RE, McNaughton N, Van MOok WNKA (2014). Using focus groups in medical education research: AMEE guide no. 91. Med Teach.

[36] Sandelowski M (2000). Whatever happened to qualitative description?. Res Nurs Health.

[37] Davis FD (1989). Perceived usefulness, perceived ease of use, and user acceptance of information technology. MIS Q.

[38] Lunney A, Cunningham NR, Eastin MS (2016). Wearable fitness technology: a structural investigation into acceptance and perceived fitness outcomes. Comput Hum Behav.

[39] Hsieh H, Shannon SE (2005). Three approaches to qualitative content analysis. Qual Health Res.

[40] Erlingsson C, Brysiewicz P (2017). A hands-on guide to doing content analysis. Afr J Emerg Med.

[41] Burnard P, Gill P, Stewart K, Treasure E, Chadwick B (2008). Analysing and presenting qualitative data. Br Dent J.

[42] Carter N, Bryant-Lukosius D, DiCenso A, Blythe J, Neville AJ (2014). The use of triangulation in qualitative research. Oncol Nurs Forum.

[43] Elnady A, Mortenson WB, Menon C (2018). Perceptions of existing wearable robotic devices for upper extremity and suggestions for their development: findings from therapists and people with stroke. JMIR Rehabil Assist Technol.

[44] Maceira-Elvira P, Popa T, Schmid AC, Hummel FC (2019). Wearable technology in stroke rehabilitation: towards improved diagnosis and treatment of upper-limb impairment. J Neuroeng Rehabil.

[45] Pino EJ, Arias Y, Aqueveque P (2018). Wearable EMG shirt for upper limb training. Conf Proc IEEE Eng Med Biol Soc.

[46] Balmain BN, Tuttle N, Bailey J, Cheng K, Duryea M, Houlihan J (2019). Using smart socks to detect step-count at slow walking speeds in healthy adults. Int J Sports Med.

[47] Horak F, King L, Mancini M (2015). Role of body-worn movement monitor technology for balance and gait rehabilitation. Phys Ther.

[48] Pak P, Jawed H, Tirone C, Lamb B, Cott C, Brunton K (2015). Incorporating research technology into the clinical assessment of balance and mobility: perspectives of physiotherapists and people with stroke. Physiother Can.

[49] Moon NW, Baker PMA, Goughnour K (2019). Designing wearable technologies for users with disabilities: accessibility, usability, and connectivity factors. J Rehabil Assist Technol Eng.

[50] van Ommeren AL, Smulders LC, Prange-Lasonder GB, Buurke JH, Veltink PH, Rietman JS (2018). Assistive technology for the upper extremities after stroke: systematic review of users’ needs. JMIR Rehabil Assist Technol.

[51] Burridge JH, Lee ACW, Turk R, Stokes M, Whitall J, Vaidyanathan R (2017). Telehealth, wearable sensors, and the internet: will they improve stroke outcomes through increased intensity of therapy, motivation, and adherence to rehabilitation programs?. J Neurol Phys Ther.

[52] Moineau B, Myers M, Ali SS, Popovic MR, Hitzig SL (2019). End-user and clinician perspectives on the viability of wearable functional electrical stimulation garments after stroke and spinal cord injury. Disabil Rehabil Assist Technol.

[53] Johansson D, Malmgren K, Alt MM (2018). Wearable sensors for clinical applications in epilepsy, Parkinson’s disease, and stroke: a mixed-methods systematic review. J Neurol.

[54] Lynch EA, Jones TM, Simpson DB, Fini NA, Kuys SS, Borschmann K (2018). Activity monitors for increasing physical activity in adult stroke survivors. Cochrane Database Syst Rev.

