# Supplementary material for: Perspectives on the prospective development of stroke-specific lower extremity wearable monitoring technology: a qualitative focus group study with physical therapists and individuals with stroke
Source: J Neuroeng Rehabil. 2020 Feb 25;17:31. doi: 10.1186/s12984-020-00666-6 (PMC7041185; doi:10.1186/s12984-020-00666-6)
Supplement: Supplementary file 1 — Additional file 1. Focus group guide for healthcare professionals. [file 12984_2020_666_MOESM1_ESM.pdf]

Development of a wearable sensor to detect lower extremity use after stroke
Focus Group Guide – Healthcare Professional

Introduction:

- Thank participants for coming and explain purpose
  - Today we would like to talk to you about your experiences treating people with lower extremity impairment and difficulty walking after stroke. We are hoping to understand if and how a wearable device that captures lower extremity movement might be useful in your practice. We are interested if such a device would be desired for assessment or home activity performance monitoring.

- Introduce self and observer
  - Moderator: The role of the moderator is to facilitate discussion and exchange of ideas between participants. This may be through promoting debate, asking open questions, ensuring everyone participants, and avoiding giving personal opinions.

  - Observer: The role of the observer is to take notes and to assist the moderator if needed with moderating the focus group process, if needed.

- Explain focus group process
  - It is a discussion between participants that is guided/facilitated by the moderator and research objective
  - Will work through the focus group guide, may go off script to explore certain topics, may prompt to stimulate discussion
  - Reminder that the focus group will be tape recorded so that it can be listened to again to more thoroughly process what was discussed
  - Information provided is confidential, will not be using real names or identifying information without permission
  - Participants do not have to come to an agreement during any of the discussions, it is open-ended and differing opinions are important for us to hear about

Part 1: Description of therapists, patient populations, and therapists' practice
"I would like to start the session by getting an overall picture of what lower extremity rehabilitation looks like for many of your clients."

- *Introductions*: Please state your name, your professional background (PT, OT, etc.), how long you have worked with stroke patients, and what setting you work in (or have worked in) where you see stroke patients

- What degree of lower extremity impairment do you typically see/treat?
  - Prompt: Is there a level of lower extremity function where it no longer becomes a treatment priority? Either too low level or too high level?

- Can you describe some examples of lower extremity goals you create with your clients?
  - Prompt: Does it generally focus on walking?
  - Prompt: Are they ever impairment-focused, i.e. strength or control-based?

- How do you generally treat lower extremity impairment and walking difficulty?
  - Prompt: Do you focus on repetitive task-based practice?
  - Prompt: Do you focus on quality (path of movement, accuracy) or quantity (repetitions, amplitude)?

- Can you provide some examples of the type of homework tasks you might assign your clients?
  - Prompt: What type of exercises or activities do you ask patients to do between sessions, how often do you want them to perform the exercise, and how many repetitions?

<u>Part 2: What aspects of LE function are clinicians interested in?</u>
We are currently working with engineers from SFU to design a wearable device for the upper extremity specifically for stroke. We are undertaking this focus group study to determine if there is a similar need or desire for a stroke-specific wearable sensor for the affected lower limb. Firstly, we would like to know what clinicians already measure, what they have difficulty measuring, and what they would like to measure.

- After stroke, what outcome measures and assessments of the lower extremity do you regularly include in your practice?
  - Prompt: Impairment-based (strength, ROM, Fugl-Meyer)?
  - Prompt: Function-based (balance, 6MWT, timed sit-to-stand)?
  - Prompt: How formally do you perform gait analysis?

- What do you feel confident measuring? What is easy to measure?

- What do you find difficult or impossible to measure on your own, that is clinically relevant to your practice?
  - Prompt: What aspects of LE function would you like to measure that you can't do on your own?
  - Prompt: Would being able to perform such a measurement affect your therapy treatment and goals? How so?

<u>Part 3: What role can wearable technology play for stroke therapists?</u>
This next section focuses on your general opinion towards wearable technology and integrating technology into therapy practice.

- How can wearable technology enhance or integrate with your practice?

- What drawbacks are there to wearable technology (from a therapeutic perspective)?

- Are you currently using available wearable technology for your practice (e.g. Fitbit, heart rate monitor)?
  - If yes, can you describe the wearable technology? Why have you chosen to use this technology?

- If no, what barriers are there keeping you from adopting current technology?
  - Prompt: Cost, ease of use, set-up time, familiarity, usefulness

- How often would you prefer to use wearable technology in your practice?
  - Only for assessments? Every session? Never?

- Would you prefer to use wearable technology therapeutically during your session or have it act as a monitor between sessions?

<u>Part 4: What would a LE wearable sensor for stroke look like to therapists?</u>

In this last section, we would like to explore how therapists envision a wearable sensor for stroke patients, from its functioning to its looks to its usability. Many commercially available sensors are not sensitive to the slower walking speeds and altered stepping patterns that are seen in stroke, resulting in reduced accuracy. It may be useful for this discussion to know that force myography is a technique that has recently been validated for the lower extremity to detect muscle activity and stepping. It registers changes in volume at a certain point in the limb, which corresponds with the underlying musculotendinous contraction.

- What do you think about a wearable device that could track leg movements and muscle contraction during gait?
  - Prompt: Would it be useful in your clinical practice?
  - Prompt: How might you use it during a therapy session?
  - Prompt: What specific leg movements are critical to categorize and measure (e.g. circumduction vs. scissoring)?

- If you were designing a wearable sensor for the lower extremity, what would you like the device to be able to do?
  - Prompt: What leg movements should it measure?
  - Prompt: Would it be helpful if it could measure specific aspects of gait (e.g. temporospatial features, kinematics?)
  - Prompt: Is there anything it shouldn't be measuring? Compensatory movements?

- If you were designing the wearable sensor, how would you design its features?
  - Prompt: What kind of user interface would it have? Would you want to view data and operate it all from a smartphone or tablet? Would you want to program and view data from a built-in display? Combination?
  - Prompt: Would you like there to be separate modes for client vs therapist?
  - Prompt: Where would you want to put the device on (i.e. ankle, hip)? How would the device look (wearable strap vs sleeve)?
  - Prompt: Is there an ideal size? How small would be too small? Conversely, how big would be too big to utilize?

- Many sensors require a calibration in order to be sensitive to its user's movements. This might involve putting the sensor on and having the subject perform several movements. How do you feel about having to calibrate the sensor prior to using it?
  - Prompt: Would this detract you from using it?

    - Prompt: What amount of calibration would you be willing to perform? What frequency and amount of time would be reasonable? (e.g. length of time, user involvement)

- What would be a reasonable price for this theoretical device?

- (Re-ask any of Part 3 if necessary (i.e. discussion was lacking), after Part 4 more concretely describes a device)

Concluding remarks

- Thank clinicians for their participation
- Provide honorarium
