# Supplementary material for: Perspectives on the prospective development of stroke-specific lower extremity wearable monitoring technology: a qualitative focus group study with physical therapists and individuals with stroke
Source: J Neuroeng Rehabil. 2020 Feb 25;17:31. doi: 10.1186/s12984-020-00666-6 (PMC7041185; doi:10.1186/s12984-020-00666-6)
Supplement: Supplementary file 2 — Additional file 2. Focus group guide for individuals with stroke. [file 12984_2020_666_MOESM2_ESM.pdf]

Development of a wearable sensor to detect lower extremity use after stroke
Focus Group Guide – Stroke Subject

Introduction:

- Thank participants for coming and explain purpose
  - Today we would like to talk to you about your experiences having leg weakness and difficulty walking due to your stroke. We are hoping to understand if and how a wearable device that captures leg movement might be useful to you and other individuals with stroke.

- Introduce self and observer
  - Moderator: The role of the moderator is to facilitate discussion and exchange of ideas between participants. This may be through promoting debate, asking open questions, ensuring everyone participants, and avoiding giving personal opinions.

  - Observer: The role of the observer is to take notes and to assist the moderator if needed with moderating the focus group process, if needed.

- Explain focus group process
  - It is a discussion between participants that is guided/facilitated by the moderator and research objective
  - Will work through the focus group guide, may go off script to explore certain topics, may prompt to stimulate discussion
  - Reminder that the focus group will be tape recorded so that it can be listened to again to more thoroughly process what was discussed
  - Information provided is confidential, will not be using real names or identifying information without permission
  - Participants do not have to come to an agreement during any of the discussions, it is open-ended and differing opinions are important for us to hear about
  - Participants should act respectfully of others, whether in agreement or disagreement of an opinion

Part 1: Description of stroke subjects and their leg weakness/walking difficulty

"I would like to start the session by getting an overall picture of how leg weakness affects your walking and day-to-day life, as well as what you are doing to manage your leg weakness and general health."

- *Introductions*: Please state your name, how long you have had your stroke, and how much walking you typically do in a day

- Please describe the weakness in your stroke-affected leg, and how it affects your walking and other day-to-day activities
  - Prompt: Is it difficult to move your leg because it feels stiff? Or does it feel weak?
  - Prompt: Can you move your leg how you want it to?
  - Prompt: Do you avoid walking because it is too troublesome? Because it is too tiring?

- Are you currently working on improving your stroke-affected leg?
  - Prompt: Are you attending physiotherapy or another treatment professional?
  - Prompt: What exercises are you doing on your own? How often, and how much?
  - Prompt: If not, why? No improvement expected? Happy with current level?

<u>Part 2: What role can wearable technology play for individuals with stroke?</u>
We are currently working with engineers from SFU to design a wearable device for the arm specifically for stroke. That device has the ability to track the arm and hand movements to count how many times the wearer reaches for something, grasps an object, or uses their hand in a day. We are undertaking this focus group study to determine if there is a desire for a similar leg sensor designed specifically for people with stroke. (Many sensors like the FitBit are not sensitive to people with leg weakness after stroke, such as slower walking speed or different movement patterns).

- Would you be interested in using wearable technology that captures leg movements in your day-to-day life?
  - Prompt: What drives your interest?
  - Prompt: How would knowing the activity of your leg be helpful to you?
  - Prompt: If not, why? Too much hassle? Uncomfortable? Aesthetic?

- What aspects of your own leg movement would you like to be able to know about?
  - Prompt: Daily step count?
  - Prompt: Time you lost balance?
  - Prompt: Quality of your leg movement? How would you define good and bad quality movement for your own leg?
  - Prompt: Distance walked?

- Why would knowing these be helpful to you?

- Do you think wearable technology would be useful for people with stroke, in general?
  - Prompt: Would having feedback increase how much walking someone performs, or their motivation to walk?
  - Prompt: If not, why?

<u>Part 3: What would a wearable sensor for the leg look like, from the perspective of people with stroke?</u>
In this last section, we would like to explore how people with stroke envision a wearable sensor for their leg, from its functioning to its looks to its usability.

- Please describe how you’ve picture the device in your mind so far?
  - Prompt: Where would you wear it?
  - Prompt: What does it look like? Is it sleek? Does it wrap around your leg or does it attach to one point?

- The wearable sensor would most likely be placed around the ankle, similar to monitors placed at the wrist. How do you feel about that?
  - Prompt: Would you still want to wear a monitor that you wore around the ankle?
  - Prompt: What thickness of the band would you be willing to wear around your ankle?
  - Prompt: Is there a better location for a wearable sensor for the leg, such as the hip or on the shoe?

- If you were designing the wearable sensor, how would you like to operate it?
  - Prompt: Would you prefer to operate it from a smartphone or tablet?
  - Prompt: Would you prefer to operate it using buttons on the device?
  - Prompt: Would you like a display on the device (like a watch) or prefer not (like an anklet)?

- (Re-ask any of Part 2 if necessary (i.e. discussion was lacking), after Part 3 more concretely describes a device)

- What would be a reasonable price if you were to pay for this device for daily use?

<u>Concluding remarks</u>

- Thank stroke subjects for their participation
- Provide honorarium
